# Climate Change, Public Health and Human Rights

**DOI:** 10.3390/ijerph192113744

**Published:** 2022-10-22

**Authors:** Benjamin Mason Meier, Flavia Bustreo, Lawrence O. Gostin

**Affiliations:** 1 Gillings School of Global Public Health, University of North Carolina at Chapel Hill, Chapel Hill, NC 27599, USA; 2 O’Neill Institute for National & Global Health Law, Georgetown Law, Washington, DC 20001, USA; 3 Fondation Botnar, 4052 Basel, Switzerland; 4 Partnership for Maternal, Newborn & Child Health, 1211 Geneva, Switzerland

## 1. Introduction

Climate change poses a cataclysmic threat to public health and human rights. Global health is inextricably linked to planetary health, with a changing climate influencing the conditions necessary for human health and safety while undermining a range of human rights. International legal agreements to mitigate emissions—from the 1992 United Nations Framework Convention on Climate Change (UNFCCC) through the 2015 Paris Agreement and into the 2021 Glasgow Climate Pact—have faced limitations in ameliorating the public health threats caused by the unfolding climate crisis. These inequitable health threats pose sweeping implications for health-related human rights, especially in low- and middle-income countries, with environmental degradation challenging the most fundamental conditions for human life and the individual rights of the most vulnerable populations. As public health concerns begin to be considered in climate change responses, human rights can provide a legal path to support international mitigation efforts and health system adaptation to address both the direct and indirect public health impacts of climate change. This Special Issue of the *International Journal of Environmental Research and Public Health* addresses the dynamic balance between global health and climate justice, bringing together policy analysis and empirical research to examine the public health threats of climate change and consider the human rights advancements necessary to frame policies for mitigation and adaptation.

In introducing the Special Issue, this editorial examines the human rights imperative to respond to the public health impacts of climate change. Part 2 introduces the role of international human rights law as a foundation for public health promotion, chronicling the long evolution of the right to health and health-related human rights to advance environmental health while examining the political neglect of public health and human rights in early climate change debates. This neglect provides the basis in Part 3 for delineating the public health threats of a changing climate and the human rights implications of those threats—including rapidly rising temperatures, pervasive air pollution, extreme weather events, infectious disease emergence, food and nutrition security, water and sanitation systems, and mental health promotion. With Part 3 ending by analyzing the human rights foundation for climate change mitigation and health system adaptation, Part 4 examines budding international efforts under the UNFCCC to mainstream human rights obligations in the global climate response. Yet despite evolving recognition of a human right to a healthy environment, international efforts within the UNFCCC Conference of the Parties have reached an impasse, with states unable to develop the legal obligations necessary to meet the catastrophic health implications of climate change. The contributors to this Special Issue grapple with this crossroads in the climate change response, with a focus on the disproportionate impacts confronting the most vulnerable populations. Bringing together international policymakers, academic researchers, and youth advocates, this Special Issue provides concrete policy proposals for future efforts, ensuring that climate change is central to the next generation of the health and human rights movement.

## 2. Human Rights Must Be Central to the Climate Change Response

Human rights law offers universal frameworks for the advancement of justice in climate change responses, clarifying international standards to establish and enforce government obligations. In codifying human rights under international law, the evolution of international human rights law has provided a foundation for legal accountability to advance public health [1]. Grounded in the right to health and rights to a wide range of underlying determinants of health—including the right to water, the right to food, the right to development, and the right to a healthy environment—a rights-based approach to the climate change response provides a means by which to operationalize international legal norms through advancements in public policy.

Policymakers have looked to the United Nations (UN) to develop and implement human rights for environmental health [2]. To build a just world order out of the ashes of World War II, governments worked through the UN General Assembly to elaborate human rights through international law, proclaiming on 10 December 1948 the Universal Declaration of Human Rights (UDHR) to create ‘a common standard of achievement for all peoples and all nations’ [3] (Preamble), detailing within it a right to ‘a standard of living adequate for . . . health and well-being’ [3] (art. 25). Building upon the UDHR, states negotiated in the ensuing years to codify specific legal obligations under two separate human rights covenants, enacting in 1966 the International Covenant on Civil and Political Rights (ICCPR) and the International Covenant on Economic, Social and Cultural Rights (ICESCR) [4,5]. These international human rights standards form the normative basis from which human rights for environmental health would evolve under international law [6].

Addressing threats to public health as ‘rights violations’, human rights have expanded dramatically to offer a legal framework for environmental health justice [7,8]. With a right to health declared for the first time in the 1948 Constitution of the World Health Organization (WHO) [9], states sought to advance public health under human rights law, establishing a human right to health in the 1966 ICESCR that would provide for ‘the right of everyone to the enjoyment of the highest attainable standard of physical and mental health’, specifically including government obligations to take all steps ‘necessary for the improvement of all aspects of environmental and industrial hygiene’ [5] (art. 12). From this foundation in the right to health, states have looked to a wide range of health-related human rights to prevent disease and promote health. This application of human rights in public health gave rise to a “health and human rights” movement, beginning in the early years of the AIDS response, expanding across a wide range of public health threats, and increasingly engaged with the global health implications of a changing climate. Yet, while health and human rights advocates have argued that adopting a rights-based approach to climate change policy can lead to more ambitious mitigation and adaptation strategies by national governments and international organizations, international environmental health debates have long neglected human rights [10,11,12,13].

Despite the catastrophic threat that climate change poses to human health and human rights—and the importance of human rights in framing climate change mitigation and adaptation—the discourse of climate change has primarily been rooted in frameworks concerning environmental protection. With this neglect extending to the human rights system, fundamental human rights instruments (including the UDHR, ICCPR, and ICESCR) do not advance rights related to environmental protection, as they were written before the rise of the global environmental movement [14].

Launching a human rights movement to protect environmental health conditions, the 1972 UN Conference on the Human Environment would provide an early conceptualization of a human right to a healthy environment. Taking place in Stockholm, this Conference provided the first international recognition of the environment as a major global concern—necessary for both individual well-being and economic development [15]. The resulting Stockholm Declaration included 26 principles, including that an individual’s environment is ‘essential to his well-being and to the enjoyment of basic human rights—even the right to life itself’ [15]. Notwithstanding this recognition of the direct role that the environment plays in realizing human rights, the Stockholm Declaration failed to call for a specific right to a safe, clean, and healthy environment. Falling short of proclaiming specific human rights, the Stockholm Declaration lacked any legally binding obligations on governments, as the instrument was designed only to bring about a ‘common outlook and common principles’ for nations.

With international climate change debates languishing in the 1980s, the Intergovernmental Panel on Climate Change (IPCC) released its first report on the changing climate in 1990, concluding that, at the current rate of human-caused greenhouse gas emissions, a 2 °C global temperature rise above preindustrial levels would be likely to occur in the decades that followed, with sweeping implications for global health [16]. In response to the IPCC report, states came together in the 1992 UN Conference on Environment and Development (UNCED) to develop the UNFCCC as a policy basis for global action. Advocates at this so-called ‘Earth Summit’ in Rio de Janeiro pushed to include human rights under the UNFCCC, building from the Stockholm Declaration’s acknowledgment of basic human rights and recognizing that ‘a right to environment would add to the protection of the biosphere whose health and safety are essential for human existence’ [17]. Although advocates in Rio argued that environmental protection cannot exist without the inclusion of human rights, states largely neglected any specific attention to human rights norms and principles. The UNFCCC sought to stabilize greenhouse gas emissions and decrease anthropogenic climate change, but it would look to subsequent Conferences of the Parties (COPs) and protocols to develop specific obligations on states parties [18].

In order to recognize the specific human rights implications of climate change, advocates continued to call on the UN to establish a clear link between human rights and safe environmental conditions. With a lack of human rights obligations in climate change mitigation debates, an international group of experts on human rights and environmental protection met in Geneva to examine the link between environmental conditions and human rights obligations. The resulting 1994 Draft Declaration of Human Rights and the Environment framed 22 principles to uphold human rights, most notably that: ‘All persons shall have the right to a secure, healthy and ecologically sound environment’. This right was seen to underlie global health, with the Draft Declaration seeking to promote ‘the right to the highest attainable standard of health free from environmental harm’ [19]. Although the UN Special Rapporteur on human rights and the environment (appointed by the Sub-Commission on Prevention of Discrimination and Protection of Minorities) would present these principles to the UN Commission on Human Rights, states declined at the time to take up this call to declare human rights for a healthy environment, holding back human rights law in climate change debates for decades.

Without human rights obligations to operationalize the goals of the UNFCCC, subsequent COPs would move closer to providing a safe and clean environment through legal obligations under the UNFCCC, but there remained an absence of human rights law in climate change governance. States continued to face shortcomings in realizing human rights through global climate change policy, as the climate continued to degrade and individuals suffered the consequences. Yet, with the rise of the field of ‘planetary health’, a recent expansion of scientific studies on the impacts of climate change on human health and human rights has provided a basis for human rights frameworks in climate change mitigation and adaptation strategies [20].

## 3. A Global Health and Human Rights Threat: Responding to Climate Change through Mitigation and Adaptation

Climate change presents a cataclysmic threat to global health and human rights. Every year, at least 150,000 premature deaths are directly linked to the climate crisis, a staggering figure that is expected to rise to 250,000 between 2030 and 2050 [21]. These impacts are being felt unequally throughout the world, with disproportionate health impacts in low-income countries and among vulnerable populations [22]. Such inequitable health harms threaten a wide range of health-related human rights. Where these human rights violations are recognized under international law, human rights can provide a normative foundation to respond to climate change, framing policies to implement climate change mitigation and adaptation to address the unjust impacts of climate change across countries and population groups.

### 3.1. The Health and Human Rights Threat of Climate Change

Climate change is impacting public health through a wide range of determinants of health, including rising temperatures, deteriorating air quality, intensifying extreme weather, emerging infectious diseases, declining nutrition, deteriorating water and sanitation, and worsening mental health, with these sweeping impacts threatening human rights that ensure the highest attainable standard of health for all.

#### 3.1.1. Rising Temperatures

As global temperatures rise and extreme heat events become more frequent, the world has seen an increase in heat-related illnesses. Extended exposure to extreme heat can cause heat exhaustion, heat cramps, heat stroke, and death [23]. Such heat exposures exacerbate preexisting conditions, such as various respiratory, cerebral, and cardiovascular diseases. These inequitable health consequences—already affecting vulnerable populations such as the elderly, children, and those with cardiovascular and respiratory diseases [24]—reflect a clear violation of the right to health, as individuals who suffer from rising temperatures are not able to protect themselves from these risks. Examining the health risks of rising temperatures, the UN Special Rapporteur on human rights and the environment has analyzed how droughts and heat waves will be longer and more frequent, leading to a higher prevalence of heat-related deaths and heat stress. The Special Rapporteur’s 2019 report concluded that ‘as global average temperatures rise, even more people’s rights will be violated, and the spectre of catastrophic runaway climate change chaos increases’, cementing certain health and human rights harms associated with climate change driven temperature increases [25]. Such health-related human rights threats, which prevent the enjoyment of the highest attainable standard of health, would not exist in the absence of climate change [26].

#### 3.1.2. Deteriorating Air Quality

These climate change impacts are the result of greenhouse gas emissions and polluting fuels, leading to a compounding risk to humans from deteriorating air quality, with several million premature deaths attributed to indoor and outdoor air pollution [27]. Beyond directly damaging air quality, these emissions contribute to additional temperature increases, which in turn increase the frequency of forest fires and other threats that further undermine air quality [28]. Ensuring clean air is fundamental to upholding public health, with air pollution linked to adverse health effects (disproportionately impacting women and children) and reflecting a failure of governments to protect the right to health [29]. The human rights basis for air quality arises out of the 1989 UN Convention on the Rights of the Child (CRC), which requires that governments ‘tak[e] into consideration the dangers and risks of environmental pollution’ [30]. Drawing from this obligation to provide salubrious environmental conditions free from pollution, the UN Special Rapporteur on human rights and the environment emphasized in 2019 that the right to breathe clean air ‘is one of the vital elements of the right to a healthy and sustainable environment’ [31]. Nations throughout the world have recognized a right to breathe air that is not harmful to health [32], yet climate change has resulted in increasing air pollution that undermines this right.

#### 3.1.3. Intensifying Extreme Weather

Undermining lives and livelihoods, climate change has further led to an increase in the frequency and severity of extreme weather events, including rising rates of drought, erosion, storms, flooding, and sea level rise, which can destroy homes and displace populations. These climate-fueled disasters have destroyed underlying determinants of health, livelihoods, and wellbeing and disrupted access to healthcare services [33]. Such catastrophic threats to public health implicate a wide range of health-related economic and social rights, including the right to health, right to housing, right to an adequate standard of living, and right to development. In threatening housing and living standards, extreme weather caused by climate change can damage homes, as settings are rendered uninhabitable through drought, erosion, and flooding, causing displacement and forced migration [34]. Where the right to development requires states to work toward the creation of an international enabling environment for development, with the benefits of development equitably shared by all, development is challenged where resources are diverted to confront the weather-related impacts of climate change [26]. In destroying entire regions and communities, these extreme weather events increasingly challenge the right to self-determination.

#### 3.1.4. Emerging Infectious Diseases

The effects of climate change on environmental degradation and weather disasters create conditions that are more favorable to new epidemics that threaten public health. Where the vast majority of emerging and re-emerging infectious diseases are zoonotic in origin, increases in animal-to-human contact have increased the risk of new outbreaks [35]. With a changing climate leading to rising temperatures and deforestation, zoonotic diseases typically found in tropical and subtropical regions are now appearing in additional regions. As seen with dengue, a mosquito-borne viral infection historically found in tropical and subtropical regions, changing climactic conditions have vastly expanded the reach of endemic dengue—from nine countries before 1970 to more than 100 countries today [36]. The right to health codifies state obligations to respond to infectious disease, with the ICESCR providing that states shall take steps necessary for the ‘prevention, treatment, and control of epidemic disease’ [5]. Avoidable infectious disease outbreaks thus undermine the right to health, limiting the availability, accessibility, acceptability, and quality of health goods, facilities, and services [37]. In addressing this threat, the UN Committee on Economic, Social and Cultural Rights has urged states to progressively realize infectious disease control through disease prevention, detection, and response efforts [38].

#### 3.1.5. Declining Nutrition

Underlying both infectious and non-communicable disease, an adequate supply of nutritious food is vital for health and achieving food security; however, climate-related weather events and droughts could lead to a ‘reduction of the quality, safety, and quantity’ of food and nutrition [39]. The Food and Agriculture Organization of the United Nations (FAO) has included ‘climate variability and extremes’ among the key drivers of global hunger and severe food crises, ‘undermining all dimensions of food security—food availability, access, utilization and stability’ [40]. Limiting adequate nutrition, climate change is projected to cause more frequent disruptions in food production and increases in food prices, with the greatest risk to children, poor populations, and tropical regions [41]. These malnutrition challenges pose a threat to key attributes of the right to food: availability, accessibility, adequacy, and sustainability [42]. The UN Special Rapporteur on the right to food has specifically examined the adverse impact of climate change on the right to food, finding that climate change will likely depress crop yields by more than five percent by 2050, with rising carbon dioxide emissions harming staple crops, reducing essential nutrients, and limiting food procurement.

#### 3.1.6. Deteriorating Water and Sanitation

Similar to the impacts on food, climate change is undermining the availability, accessibility, and quality of water and sanitation, with drought and pollution deteriorating drinking water and water for domestic use. The IPCC has concluded that the competition for water will intensify as climate change reduces renewable surface water and groundwater resources in most dry subtropical regions [43]. Climate change is expected to increase the risk of water scarcity in urban areas, alongside major impacts on water availability and supply in rural areas. Disproportionally impacting individuals in vulnerable situations, this reduction in the quantity and quality of water will violate the human right to water. Where the UN Committee on Economic, Social and Cultural Rights has recognized that the human right to water ‘entitles everyone to sufficient, safe, acceptable, physically accessible and affordable water for personal and domestic uses’ [44], the rights to water and sanitation require that all states progressively realize safe, clean, accessible, and affordable drinking water and sanitation for all [45]. Affirmed by the UN General Assembly and Human Rights Council, the UN Special Rapporteur on the rights to water and sanitation has addressed the ways in which the rights to water and sanitation of people in vulnerable situations (specifically persons living in poverty, indigenous people, women and girls, children, persons with disabilities, migrants and displaced persons, older persons, and ethnic minorities) will suffer inequitably from climate change due to droughts, floods, deglaciation, and more [46].

#### 3.1.7. Worsening Mental Health

These catastrophic challenges are creating stressors that worsen the mental health of individuals throughout the world. Recurring climate disasters, including the aftermath of hurricanes and wildfires, present direct stressors, compounded by indirect stressors through worsening living conditions. Individuals are already experiencing climate-related psychological distress, depression, and post-traumatic stress at unprecedented rates throughout the world, with evidence highlighting how adolescents and young people are increasingly facing “climate anxiety” and “eco-anxiety” as they consider the uncertainties in their future [47,48]. With environmental changes leading to a loss of control, this insecurity will bring about higher instances of aggression, violence, hopelessness, and depression [49]. These climate-driven mental health harms directly violate the right to health, and in recognizing mental health as a central part of the right to health, the UN Special Rapporteur on the right to health has concluded that: ‘Everyone, throughout their lifetime, requires an environment that supports their mental health and well-being’ [50]. Climate change threatens these central mental health aspects of the right to health, diminishing the highest attainable standard of mental health and social wellbeing.

### 3.2. The Human Rights Foundation for the Climate Change Response

Given these human rights threats posed by a changing climate, human rights norms and principles provide a path to respond to these cataclysmic health conditions, framing global efforts to mitigate a warming world and national efforts to adapt to the public health impacts.

#### 3.2.1. Climate Change Mitigation

Human rights obligations provide a foundation to frame efforts that governments must take to mitigate climate change. Where mitigation measures seek to reduce greenhouse gas emissions, avoiding temperature increases and the resulting health harms, states will need to strengthen regulations on the most polluting sectors. Even the health sector must be reformed, with evidence showing that, if it were a country, the health sector would be the world’s fifth-largest carbon emitter [51]. The UN Human Rights Committee first recognized mitigation as a human rights obligation in 2018: ‘Environmental degradation, climate change and unsustainable development constitute some of the most pressing and serious threats to the ability of present and future generations to enjoy the right to life’ [52]. From this focus on the right to life, five UN human rights treaty bodies issued a rare joint statement in 2019 to call on states to consider their human rights obligations in mitigating the threat of climate change. In this joint statement, the treaty bodies reviewed the sweeping impacts of climate change across a wide range of rights, and concluded that failing to act to prevent foreseeable human rights harm caused by climate change could ‘constitute a violation of States’ human rights obligations’ [53].

This framing of climate change mitigation as a human rights obligation provides a pathway for international action to prevent the most catastrophic impacts of climate change. The public health threats of climate change violate a wide range of human rights, and opportunities must be taken across states to mitigate these threats to protect human rights [54]. With climate change undermining human rights for generations to come, the UN Committee on the Rights of the Child declared in 2021 that ‘a state can be found responsible for the negative impact of its carbon emissions on the rights of children both within and outside its territory’ [55]. This decision under the CRC holds that states bear cross-border responsibilities for the harms of climate change, requiring states not only to ensure that their measures do not accelerate climate change but to dedicate the maximum available resources to progressively realizing mitigation measures. Under this extraterritorial obligation, states must not accelerate climate change and must take steps to mitigate this global threat—for which they bear cross-border responsibility to the entire global community and to future generations—raising a human rights imperative for states to work together through global governance to effectively face the global threat of climate change [56].

In pursuing such climate change mitigation policies to realize human rights obligations, human rights frameworks can ensure that government measures taken to mitigate climate change do not themselves violate human rights. Developing this “rights-based approach” to mitigation efforts, states agreed at the 2010 Cancun climate summit that governments ‘should, in all climate change-related actions, fully respect human rights’ [57]. To respect human rights while mitigating climate change, a rights-based approach to development will be necessary, incorporating into mitigation efforts a wide range of cross-cutting human rights principles: universality and inalienability, indivisibility, interdependence and interrelatedness, non-discrimination and equality, participation and inclusion, accountability, and the rule of law [26,58]. For example, climate change mitigation measures have been undertaken in several countries without the meaningful engagement and participation of indigenous peoples, even when mitigation projects take place on indigenous land—as often seen in the mining of alternative energy resources, forest conservation, tree-planting projects, or resettlement schemes [59]. These prospective threats to human rights demand that affected individuals and communities participate in mitigation policy decision making, without discrimination in the design and implementation of mitigation efforts, and with access to due process for addressing community concerns and to appropriate remedies if rights are violated [26].

#### 3.2.2. Climate Change Adaptation

Yet, in recognizing the limitations of mitigation efforts to prevent many of the public health harms of climate change, governments must also implement adaptation laws, policies, and programs that enable populations to respond to the likely health impacts. Such adaptation will require sustainable and resilient health systems, infrastructures, and technologies [60]. The health sector is particularly vulnerable to the impacts of climate change, and governments must reform health systems to predict, prepare for, and respond to the public health consequences. Supporting this global health imperative, human rights provide a framework, at the national level and through international assistance, ‘to ensure that all persons have the necessary capacity to adapt to climate change’ [26].

Human rights provide a foundation to frame necessary health systems reforms. Health systems can adapt to climate change risks by reducing public health vulnerabilities and developing specific system capacities to understand how climate change affects specific populations and service delivery. These reforms can be framed under the human right to health, which, in accordance with the ICESCR, requires ‘the creation of conditions which would assure to all medical service and medical attention in the event of sickness’ [61] (art. 12.2(d)), assessing the response of health systems on the basis of their:

Availability—Health systems should adapt in ways that ensure a sufficient number of functioning facilities, goods, services, and programs to address the health harms of climate change.

Accessibility—Health systems must ensure four overlapping dimensions of accessibility, including (1) accessibility without discrimination; (2) physical accessibility of goods, services, facilities, and determinants, safely within reach, despite geographical challenges, population displacements, and weather patterns; (3) economic accessibility through affordable goods and services; and (4) information accessibility, allowing all individuals to seek, receive, and impart information concerning health, including information regarding the health implications of climate change [62].

Acceptability—Health interventions must be acceptable not only to individual beneficiaries but must also be ethically and culturally appropriate, including special emphasis on ensuring acceptability for those most likely to be impacted by, and therefore vulnerable to, climate change.

Quality—The climate change response will require scientifically and medically appropriate services and technologies, including in measures for protection against climate change and response to health harms [61].

These health system reforms to uphold the right to health amid the rising challenges of climate change must take special care to protect vulnerable populations. Climate change will pose particular health risks to vulnerable populations that have long faced inequitable health challenges (including older persons, younger persons, incarcerated populations, persons with substance use disorders, and those with disabilities), and additional populations will face vulnerability as a result of climate change (including populations in low-lying areas and informal settlements) [63,64]. Human rights principles of equality and non-discrimination can inform health sector reforms and adaptation efforts to address these vulnerabilities, ensuring that health systems are prepared to support the needs of all populations equitably amid the inequitable health threats of a changing climate [26]. Examined across countries, this inequity will also be felt globally, where climate change’s impact on health systems is being experienced disproportionately in low- and middle-income countries, which often face challenges in responding quickly through their national health systems. Responding to this global inequity, global health governance must play a key role in coordinating international assistance and cooperation to support health system adaptation, with high-income nations (which have long profited economically through actions that have led to the current climate crisis) bearing extraterritorial obligations to support less-wealthy countries in the climate change response [65]. Central to this global governance response, the WHO must facilitate international support for national health systems, providing guidance to national ministries of health and their partners to design comprehensive responses to public health challenges under climate change policy [66].

## 4. Rising Efforts to Mainstream Human Rights in Climate Change Policy

Despite the historical limitations of the UNFCCC to integrate health-related human rights, global governance institutions have increasingly begun to address the challenges of a changing climate for public health and human rights.

To draw attention to the public health consequences in global health governance, the WHO devoted its 2008 World Health Day to climate change, raising awareness of climate change as a global health challenge. This theme was developed to recognize the global health security threats posed by climate change, including the rise of infectious disease outbreaks and intense natural disasters [67]. The WHO emphasized that climate change will impact the entire planet, but that certain people will bear a disproportionate vulnerability to the health effects, with climate change ‘affect[ing] some of the most fundamental determinants of health: air, water, food, shelter, and freedom of disease’ [68]. Looking to the right to health as a basis of concern, the WHO concluded that ‘human health needs to be placed at the centre of environment and development decisions’ as nations seek climate change mitigation and adaptation under the UNFCCC [68].

Human rights governance would also take up these climate change concerns, with the Office of the UN High Commissioner for Human Rights (OHCHR) conducting a 2009 study on the relationship between human rights and climate change, concluding that:

Climate change impacts, directly and indirectly, an array of internationally guaranteed human rights. States (duty-bearers) have an affirmative obligation to take effective measures to prevent and redress these climate impacts, and therefore, to mitigate climate change, and to ensure that all human beings (rights-holders) have the necessary capacity to adapt to the climate change crisis [69].

The following year, the UNFCCC COP in Cancun recognized for the first time the relationship between human rights and climate change, with states agreeing that ‘[p]arties should, in all climate change-related actions, fully respect human rights’ [70]. The OHCHR followed up on this international consensus in a 2011 *Analytical Study on the Relationship Between Human Rights and the Environment,* which examined why and how human rights can be used to ensure environmental protection, including in the case of climate change [71].

While the failure to adopt a right to a clean and safe environment under international law continued to limit human rights implementation under the UNFCCC, states continued to seek legally binding obligations to lower CO_2_ emissions in an effort to facilitate cleaner air, improved environmental conditions, and lower temperatures. Recognizing the catastrophic implications of a deteriorating climate, COP21 in Paris sought to hold ‘the increase in global average temperature to well below 2 °C above pre-industrial levels’ [72]. In developing the 2015 Paris Agreement, activists pushed for the inclusion of core principles of human rights in climate change policy. Supporting these calls from activists, the WHO convened 30 ministers of health to advance global health in the Paris Agreement through the human right to health [73]. Despite this rising human rights advocacy and WHO support for the right to health in Paris, key states (including the United States, Norway, and Saudi Arabia) worked to block the inclusion of human rights language and obligations [74], limiting health and human rights in the final Paris Agreement to the preambular recognition that states must ‘promote and consider their respective obligations on human rights [and] the right to health’ [72]. Yet, with this initial recognition of the right to health under the UNFCCC, states developed provisions on adaptation measures to combat the health impacts of climate change through the development of climate-resilient systems. The Paris Agreement would reflect the most comprehensive and inclusive climate change agreement to date; however, it would not be sufficient to mitigate the rising threat, failing to keep projected temperatures below 2 °C and exposing the risk of a global temperature rise above 3 °C [75].

Understanding the public health and human rights implications of a rise in global temperatures above 2 °C, the WHO continued to engage in climate change debates following the Paris Agreement. In moving to address health-related human rights, WHO Director-General Margaret Chan addressed the Human Rights Council in 2016, arguing that centering a human rights approach offered the most impactful point of entry to hold nations accountable to health obligations under the Paris Agreement and concluding that ‘human rights obligations, standards, and principles have the power to shape policies for climate change mitigation and adaptation’ [76]. The WHO furthered the debate on the links between climate change and human rights in the 2018 Global Conference on Air Pollution and Health, advancing discussion of a human right to breathe clean and healthy air [77]. With WHO Director-General Tedros Adhanom Ghebreyesus describing clean air as a human right [78], recognizing that few states have prioritized plans to adapt to climate change, the WHO sought to provide guidance on how nations can implement climate-resilient health systems, advising states to develop surveillance and early-warning systems to address health outcomes that are inextricably linked to a changing climate [62].

As the WHO pushed to integrate health into climate change policy, a right to a healthy environment would begin to take shape under international law. The UN Special Rapporteur on human rights and the environment examined a right to breathe clean air within the larger right to a healthy environment, acknowledging the potential violation of a right to a healthy environment when states fail to address climate change [25]. Yet, the COPs after the Paris Agreement added little to global health policy in realizing human rights in the face of a changing climate. Given the failure of climate change policy to operationalize human rights, advocates moved to bring recognition to a needed right of a healthy environment in the UN human rights system [79]. Looking to the UN Human Rights Council, states and advocates sought to declare a right to a healthy environment [80]. In advancing the right to a healthy environment, and the rights that make up a healthy environment, as essential for human life and dignity [81], the Human Rights Council unanimously resolved in late 2021 to recognize a right to a safe and healthy environment. The WHO looked to this human rights debate in considering the larger range of rights threatened by climate change, with the WHO introducing a ‘right to clean air’ under its Global Air Quality Guidelines [80].

On the heels of this consequential first step by the UN Human Rights Council, delegates from across the globe gathered in Glasgow for COP26 in November 2021. States in Glasgow sought to slash emissions by 2030, seeking for all nations to be carbon-neutral by the middle of the century [82]. This would be the most decisive mitigation policy ever developed to keep global temperature rise below 1.5 °C, yet there remained little mention of human rights. In considering the importance of human rights in mitigation policy, the UN High Commissioner for Human Rights Michelle Bachelet urged states to seek a ‘sustainable, zero-carbon economy’, concluding: ‘This is a human rights obligation and a matter of survival. Without a healthy planet to live on, there will be no human rights—and if we continue on our current path—there may be no humans’ [83]. This human rights advocacy extended to climate change adaptation, with the head of the UK Environment Agency urging immediate adaptation efforts as a human rights imperative: ‘While mitigation might save the planet, it is adaptation, preparing for climate shocks, that will save millions of lives’ [84]. Despite this robust advocacy, the Glasgow Agreement saw only one perfunctory mention of human rights. Amnesty International spoke for many activists in concluding that they were ‘extremely disappointed that the COP26 outcomes present only minimal and incremental progress to protecting human rights in…the climate crisis’ [85].

Even as COP26 presented the strongest language ever seen in any climate document, with 151 nations signing on to ‘phase down’ coal consumption and other CO_2_ emissions [86], activists left Glasgow disappointed by the limited acknowledgment of health and human rights. Climate change policy would continue to require a just approach that addresses the needs of the most vulnerable and an accountability framework that facilitates state action for mitigation and adaptation, but states would need to look beyond climate change policy to advance health-related human rights. Drawing from the 2021 debates in the UN Human Rights Council, the UN General Assembly took up this human rights debate on 28 July 2022, declaring access to a clean, healthy, and sustainable environment to be a universal human right. This General Assembly resolution recognizes the right to a clean, healthy, and sustainable environment as ‘related to other rights and existing international law’, affirming that its promotion ‘requires the full implementation of multilateral environmental agreements under the principles of international environmental law’. It calls upon all stakeholders ‘to adopt policies, to enhance international cooperation, strengthen capacity-building and continue to share good practices in order to scale up efforts to ensure a clean, healthy and sustainable environment for all’ [87].

This resolution recognizing a human right to a healthy environment, the culmination of a 50-year effort arising out of the Stockholm Declaration, raises new international political support to uphold environmental health and human rights obligations in climate change debates [88]. While some delegations continue to find a lack of a universally agreed-upon scope and context for the right to a clean, healthy, and sustainable environment, describing the resolution as a ‘political statement, not a legal affirmation by the Assembly’ [89], the unanimous vote of the General Assembly (with 161 votes in favor and 8 abstentions) will assist states in collectively advancing the realization of environmental health and human rights responsibilities in future COP debates. This political assertion of a right to a healthy environment reflects an important step in protecting planetary health and serves as a catalyst for environmental justice—in COP27 and beyond. As the struggle continues to introduce legally binding norms to enshrine a human right to a healthy environment in climate change policy, a foundation has been established to bring together climate change, public health, and human rights.
